# Leukemia risk assessment of exposure to low-levels of benzene based on the linearized multistage model

**DOI:** 10.3389/fpubh.2024.1355739

**Published:** 2024-05-14

**Authors:** Kexin Jin, Fukang Zhu, Bo Wu, Minyan Li, Xue Wang, Xiurong Cheng, Ming Li, Deyin Huang, Caihong Xing

**Affiliations:** ^1^State Key Laboratory of Trauma and Chemical Poisoning, National Institute for Occupational Health and Poison Control, Chinese Center for Disease Control and Prevention, Beijing, China; ^2^Chinese Academy of Inspection and Quarantine, Beijing, China; ^3^Institute of Occupational Health, Tianjin Bohai Chemical Industry Group Co. Ltd., Tianjin, China; ^4^Occupational Health Monitoring and Evaluation, Department of Jinan Railway Disease Control and Prevention Center, Jinan, China

**Keywords:** benzene, multi-stage modeling, risk assessment, S-PMA, t, t-MA

## Abstract

**Objectives:**

To assess leukemia risk in occupational populations exposed to low levels of benzene.

**Methods:**

Leukemia incidence data from the Chinese Benzene Cohort Study were fitted using the Linearized multistage (LMS) model. Individual benzene exposure levels, urinary S-phenylmercapturic acid (S-PMA) and trans, trans-muconic acid (*t, t*-MA) were measured among 98 benzene-exposed workers from factories in China. Subjects were categorized into four groups by rounding the quartiles of cumulative benzene concentrations (< 3, 3–5, 5–12, ≥12 mg/m^3^·year, respectively). The risk of benzene-induced leukemia was assessed using the LMS model, and the results were validated using the EPA model and the Singapore semi-quantitative risk assessment model.

**Results:**

The leukemia risks showed a positive correlation with increasing cumulative concentration in the four exposure groups (excess leukemia risks were 4.34, 4.37, 4.44 and 5.52 × 10^−4^, respectively; *P*_trend_ < 0.0001) indicated by the LMS model. We also found that the estimated leukemia risk using urinary *t, t*-MA in the LMS model was more similar to those estimated by airborne benzene compared to S-PMA. The leukemia risk estimated by the LMS model was consistent with both the Singapore semi-quantitative risk assessment model at all concentrations and the EPA model at high concentrations (5–12, ≥12 mg/m^3^·year), while exceeding the EPA model at low concentrations (< 3 and 3–5 mg/m^3^·year). However, in all four benzene-exposed groups, the leukemia risks estimated by these three models exceeded the lowest acceptable limit for carcinogenic risk set by the EPA at 1 × 10^−6^.

**Conclusion:**

This study demonstrates the utility of the LMS model derived from the Chinese benzene cohort in assessing leukemia risk associated with low-level benzene exposure, and suggests that leukemia risk may occur at cumulative concentrations below 3 mg/m^3^·year.

## 1 Introduction

Benzene is an organic solvent widely used in the synthesis of organic chemicals such as fibers, rubbers, and pesticides. Occupational exposure to benzene may occur in the chemical industry for the synthesis of a great variety of chemical products, in the production and handling of gasoline fuels, and in the production and use of solvents and thinners for paints, lacquers, adhesives, glues, plastics, rubber. Other workers who may be exposed to benzene because of their occupations include steelworkers, printers, shoemakers, laboratory technicians, firefighters, and gas station employees. The International Agency for Research on Cancer (IARC) has classified benzene as carcinogenic to humans, giving rise to acute myeloid leukemia (AML), and possibly increasing the risk of acute lymphoblastic leukemia (ALL), chronic lymphocytic leukemia (CLL), multiple myeloma (MM), and non-Hodgkin lymphoma (NHL) (1). The occupational exposure limit (OEL) for benzene has been set at 3 mg/m^3^ (0.92 ppm) in China, and the US ACGIH has lowered the Threshold limit value (TLV) to 0.02 ppm (2). It has been reported that workers exposed to less than 1 ppm of benzene may still experience adverse health effects. Lan et al. (3) reported that the total white blood cell (WBC) and platelet counts were significantly lower in workers exposed to benzene at 1 ppm than in the unexposed group. In our previous study, we found a significantly increased frequency of aneuploidy in sperm cells of male workers exposed to 1 ppm benzene compared to controls (4). An evidently elevated risk of myelodysplastic syndromes (MDS) for petroleum distribution workers exposed to >2.93 ppm-years of benzene exposure as compared to those exposed to <0.348 ppm-years, OR = 4.33 (95% CI = 1.31–14.3) (5). Stenehjem et al. (6) found a significant dose response for AML from cumulative benzene exposures just below 1 ppm-year and the same for multiple myeloma. In addition, Talbott et al. (7) demonstrated an 11-fold risk of AML from cumulative benzene exposures well below 1 ppm-year. Therefore, the study of quantitative leukemia risk at low levels of benzene exposure (<3 mg/m^3^·year) is of great value.

The monitoring of low-level benzene exposure includes air concentration monitoring and biological exposure monitoring. At present, the United States, Germany, Spain, and other countries have successively used urine *t, t*-MA and S-PMA as biological monitoring indicators of occupational benzene exposure (8).

The dose–response relationship between exposure to chemicals and the probability of cancer occurrence has been assessed by cancer models such as the linearized multistage (LMS) model, the one-hit model, probit model, and Weibull model (9, 10). With the development of methods for assessing leukemia risk, the USEPA and the National Institute for Occupational Safety and Health (NIOSH) jointly developed the benchmark dose software (BMDs) (11) for obtaining the LMS models in observational epidemiologic studies (12). The LMS model is the most widely used by the USEPA in dose–response assessment model (13). Therefore, we used this model to assess the risk of leukemia associated with occupational exposure to low levels of benzene. The LMS model was fitted to leukemia incidence data from the Chinese Benzene Cohort Study (14) to assess leukemia risk based on external (airborne benzene) and internal (urinary S-PMA and *t, t*-MA) benzene exposure concentrations. The reliability of the LMS model results was also validated using the EPA model and the Singapore semi-quantitative risk assessment model.

## 2 Materials and methods

### 2.1 The linearized multistage model

The linearized multistage (LMS) model (15) expressed as Equation (1) has been used to conduct a cancer risk assessment:


(1) $$P\left(d\right)=r+\left(1-r\right)\left[1-\exp\left(-\overset{\infty }{\underset{i=0}{\sum }}a_{i}d^{i}\right)\right]$$

where *d* represents a lifetime-exposure to carcinogenic chemicals. *P(d)* is the excess cancer risk (probability of the occurrence of cancer) during an individual’s lifetime when exposed to a carcinogenic chemical at dose *d*; *i* (> 0) is the number of stages. Both *r* and *a_i_* are parameters to be estimated in the LMS model, where *r* is the background response level.

Cumulative exposure was considered as lifetime-exposure in the dose–response models (16). To estimate the leukemia risk from exposure to benzene, the LMS model based on cumulative exposure can be expressed as Equation (2):


(2) $$P\left(D_{c}\right)=r+\left(1-r\right)\left[1-\exp\left(-\overset{\infty }{\underset{i=0}{\sum }}a_{i}{D_{c}}^{i}\right)\right]$$

where *Dc* represents the cumulative exposure of benzene. Since the cumulative exposure was calculated by the multiplying time-weighted average concentration (*C_TWA_*) and the working duration (*T*) (17), the LMS model can be finally expressed as Equation (3), by which the leukemia risk from *T* years of exposure to benzene at a dose of *C_TWA_* can be estimated.


(3) $$P\left(D_{c}\right)=r+\left(1-r\right)\left\{1-\exp\left[-\overset{\infty }{\underset{i=0}{\sum }}a_{i}{\left(C_{TWA}\right)}^{i}T^{i}\right]\right\}$$

### 2.2 Parameter estimation of the LMS model

The parameters (*r* and *a_i_*) of the LMS model were estimated using the maximum likelihood estimates (MLEs) method in the BMDs based on the data from the Chinese benzene cohort study. In this benzene study, a cohort of 74, 828 benzene-exposed and 35,805 unexposed workers employed from 1972 to 1987 in 12 cities in China was identified, and the incidence of hematologic neoplasms was determined (14). This study is particularly suitable to fit the LMS model for assessing the dose–response effects between benzene and leukemia due to its large sample size, long follow-up time, specific exposure to benzene, and accurate exposure estimates.

### 2.3 EPA model

The EPA model estimates a worker’s lifetime risk of cancer based on the chemical’s Inhalation Unit Risk (IUR) and Exposure Concentration (EC) with the Equation (4):


(4) Risk=IUR×EC

In the above equation, *Risk* is the lifetime cancer risk; *IUR* is the inhalation unit risk (μg/m^3^), which refers to the upper estimate value of life-long cancer risk resulting from continuous exposure of air to a toxic and hazardous chemical concentration of 1 μg/m^3^. According to the EPA IRIS database, the *IUR* for benzene-induced leukemia was found to be (2.2–7.8) × 10^−6^ (μg/m^3^)^-1^. *EC* is the lifetime average exposure concentration (μg/m^3^), and *EC* is calculated by Equation (5):


(5) $$EC=\left(CA\times ET\times EF\times ED\right)/AT$$

where *CA* is the concentration of toxic and hazardous chemicals in the air of the workplace (μg/m^3^); *ET* is the exposure time per day (h/d); *EF* is the exposure frequency of employees in the workplace (d/y); *ED* is the duration of exposure during the exposure period (years); and *AT* is the average exposure time (h), the value of which is life expectancy × 365 d × 24 h. In 2020, the *per capita* life expectancy in China was 77.93 years.

### 2.4 Singapore semi-quantitative risk assessment model

The risk level can be calculated by the equation: $\mathrm{Risk}=\sqrt{HR\times ER}$ , where *HR* represents the hazard rating, and *ER* represents the exposure rating. *HR* can be classified into 1 ~ 5 levels according to the toxicity, irritation, mutagenicity, teratogenicity, and carcinogenicity of chemicals. Benzene is a confirmed human carcinogen, so the *HR* is 5. *ER* is calculated by the ratio of workers’ exposure dose (*E*) to the occupational exposure limit (*OEL*) of benzene, *ER = E/OEL*. When *E/OEL*<0.1, *ER* = 1; 0.1 ≤ *E/OEL*<0.5, *ER* = 2; 0.5 ≤ *E/OEL*<1.0, *ER* = 3; 1.0 ≤ *E/OEL*<2.0, *ER* = 4; *E/OEL* ≥ 2.0, *ER* = 5. The calculated *Risk* values were rounded up and graded: 1 for negligible risk; 2 for low risk; 3 for medium risk; 4 for high risk; 5 for very high risk (18).

### 2.5 Study population and exposure assessment

A total of 103 benzene-exposed workers were recruited from three factories in Shandong and Anhui province, China. All these factories used benzene-containing adhesives and raw materials. Protocols, questionnaires, and consent forms were reviewed and approved by the Committee for the Protection of Human Subjects at the National Institute for Occupational Health and Poison Control, Chinese Center for Disease Control and Prevention (China CDC). The ethics approval number is 201812. All workers signed a consent form. Information on working duration, cigarette smoking, and diet habits was collected in the questionnaire.

Benzene-exposed workers wore a personal passive air monitor (SKC VOC 575–002) near their breathing zone for a whole 8-h workday and provided a spot urine at the end of the work shift. Passive air monitors were transported at room temperature, while urine samples were transported in dry ice to the China CDC. Each participant was monitored 2–3 times over a six-month period.

Air monitors were desorbed with 1 mL of carbon disulfide for 30 min and analyzed for benzene using gas chromatography with flame ionization detection (Agilent 7890A). Creatine concentration in urine (μg/g Cr) was analyzed via liquid chromatography. S-PMA and *t, t*-MA were detected by high-performance liquid chromatography/tandem mass spectrometry (19, 20).

### 2.6 Statistical analysis and data processing

Data normality was assessed by the Kolmogorov–Smirnov test. As most variables had a skewed distribution, the correlation between urinary metabolites (S-PMA and *t, t*-MA) and air benzene was analyzed by the non-parametric Mann–Whitney test. Test for trend was performed by ANOVA trend analysis. To obtain an approximate normality, concentration of S-PMA and *t, t*-MA were ln-transformed. Multiple linear regression analyses were used to estimate the relationship between urinary metabolites and air benzene, adjusting for cigarette smoking (smoking was coded as a dummy variable: smoker = 1, non-smoker = 0). Statistical analyses were performed using SAS software (Version 9.4; SAS Institute, Cary, NC, United States). The parameters of the LMS model were estimated by benchmark dose software (BMDS 3.32 version).

## 3 Results

### 3.1 The LMS model based on the Chinese benzene cohort study

In the Chinese benzene cohort study (14), leukemia risk increased with increasing cumulative benzene exposure. For workers with cumulative benzene exposure below 130 mg/m^3^·year (median 65 mg/m^3^·year), the RR for leukemia is 0.81 (95% CI = 0.38 to 1.68), and the leukemia incidence is 4.07 × 10^−4^; workers with cumulative benzene exposure of 130 to 646 mg/m^3^·year (median 388 mg/m^3^·year), the RR for leukemia is 2.02 (95% CI = 1.09 to 3.82), and the leukemia incidence is 10.16 × 10^−4^ (Table 1). Using the data from the Chinese benzene cohort study (entering the three sets of cumulative exposure concentration midpoints, total population size, and number of leukemia cases into the BMDs), the parameters (r and ai) of the LMS model were obtained through the BMDs. The results show that the goodness-of-fit was best when *i* was 1 (*p* = 0.25), and the parameter *a1* was 1.38 × 10^−6^, the background response level *r* was 4.32 × 10^−4^. Hence, we obtained the LMS model for risk assessment of benzene-induced leukemia:

**Table 1:** Dose–response relationship between benzene exposure and leukemia in Chinese cohort from Linet et al. studies (14).

Group	Midpoint of cumulative exposure (mg/m^3^·year)	Total numbers	Leukemia cases	RR (95% CI)	Leukemia incidence
unexposed subjects	0	35, 804	18	1	5.02 × 10^−4^
< 130 mg/m^3^·year	65	31, 923	13	0.81 (0.38–1.68)	4.07 × 10^−4^
130–646 mg/m^3^·year	388	24, 605	25	2.02 (1.09–3.82)	10.16 × 10^−4^


(6) $$\begin{array}{c} P\left(D_{c}\right)=4.32\times 10^{-4}+\left(1-4.32\times 10^{-4}\right)\\ \left[1-\exp\left(-1.38\times 10^{-6}C_{TWA}\times T\right)\right] \end{array}$$

### 3.2 The LMS model based on urinary benzene metabolites concentration

We observed that among the 103 workers exposed to benzene, a total of 5 individuals exhibited air benzene values below the Limit of Detection (LOD = 0.05 mg/m^3^). The correlation between urine benzene metabolites and air benzene concentration was studied in 98 benzene exposed workers with air benzene value > LOD. Out of 98 benzene exposed workers included 23 (23.5%) smokers and 75 (76.5%) were nonsmokers. The concentrations of benzene exposure, both internal and external, were monitored three times for workers (some people lost to follow-up). Urine samples were excluded if the urine creatinine concentration was <0.3 g/L or > 3 g/L, or if the air benzene concentration was less than 3 mg/m^3^ while *t, t*-MA exceeded 1,000 μg/g Cr. A total of 217 valid samples (each includes air benzene, urine S-PMA, and *t, t*-MA) were obtained. The median concentrations of air benzene, urine S-PMA, and *t, t*-MA were 3.53 mg/m^3^, 6.83 μg/g Cr, and 219.12 μg/g Cr, respectively. The two urinary benzene metabolites, S-PMA and *t, t*-MA, were significantly correlated with air benzene after adjusting for cigarette smoking (SMO; ln-transformed values; *r* = 0.76 and *r* = 0.67, respectively).

**Figure 1:**
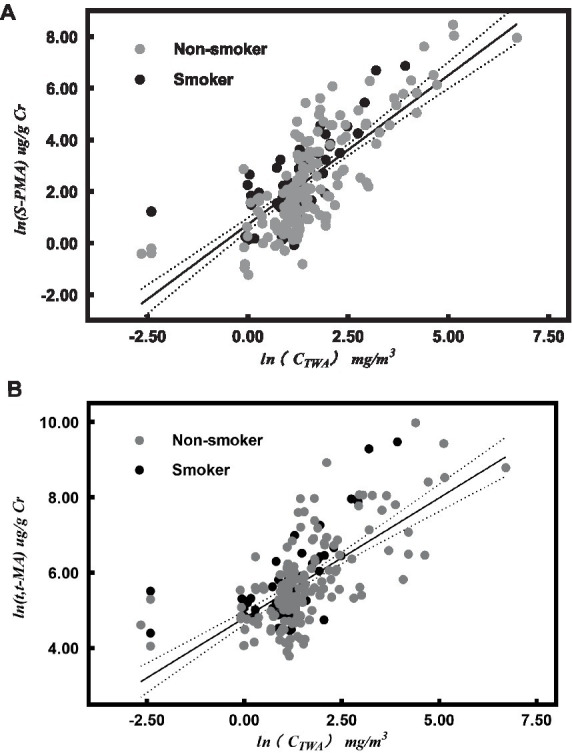
Correlation between urinary metabolites [urinary S-PMA **(A)** and *t, t*-MA **(B)**] and air benzene concentrations (ln-transformed values) after adjusting for cigarette smoking (SMO). The dark full line represents the regression line, and the dotted lines represent the 95% confidence interval for individual results in both **(A,B)**.

*ln(C_TWA_)* was linear associated with *ln*(*S-PMA*; R^2^ = 0.58, *p* < 0.001; Figure 1A):


(7) $$\ln\left(S-PMA\right)=0.77\ln\left(C_{TWA}\right)+0.12SMO+0.54$$

*ln(C_TWA_)* was linear associated with *ln*(*t, t-MA*; R^2^ = 0.45, *p* < 0.001; Figure 1B):


(8) $$\ln\left(t.t-MA\right)=0.68\ln\left(C_{TWA}\right)+0.08SMO+4.73$$

According to the correlation between air benzene and urinary metabolites, Equation (7) and Equation (8) were substituted into Equation (6) to obtain Equations (9) and (10) for evaluating the risk of leukemia based on S-PMA and *t, t*-MA values:


(9) $$\begin{array}{l} P\left(S-PMA\right)=4.32\times 10^{-4}+\left(1-4.32\times 10^{-4}\right)\;\\ \;\times \left\{1-\exp\left[-1.38\times 10^{-6}\begin{array}{l} {}[\exp[(\ln\left(S-PMA\right)\\ -0.12SMO-0.54 \end{array})/0.77\right]]T]\right\} \end{array}$$

(10) $$\begin{array}{l} P\left(t,t-MA\right)=4.32\times 10^{-4}+\left(1-4.32\times 10^{-4}\right)\\ \;\times \left\{1-\exp\left[-1.38\times 10^{-6}\begin{array}{l} {}[\exp[(\ln\left(t,t-MA\right)\\ -0.08SMO-4.73 \end{array})/0.68\right]]T]\right\} \end{array}$$

### 3.3 Leukemia risk assessment

The average duration of exposure for the 98 participants with detectable exposure to benzene was 1.78 years (range: 0.08 to 7.83 years). Subjects were categorized into four groups based on the rounded quartile of the cumulative benzene airborne level (< 3, 3–5, 5–12, ≥12 mg/m^3^·year; Table 2). As shown in Table 3, the excess risk of leukemia increased with increasing air benzene exposure (*P*_trend_ = 0.0001). The excess leukemia risks for workers exposed to <3, 3–5, 5–12, and ≥ 12 mg/m^3^·year (mean: 25.73 mg/m^3^·year) were 4.34, 4.37, 4.44, and 5.52 × 10^−4^, respectively, all exceeding the EPA’s lower bound value for acceptable carcinogenic risk (1 × 10^−6^). The results also indicated that the leukemia risks estimated from S-PMA and *t, t*-MA were slightly higher than those estimated from air benzene concentrations across all four concentration groups. Moreover, the risk estimated derived from *t, t*-MA was closer to those obtained from air benzene concentrations.

**Table 2:** Summary of three benzene exposure measurements for benzene-exposed workers.

		Working duration (year)	Air benzene (mg/m^3^)	S-PMA (μg/g Cr)	*t, t*-MA (μg/g Cr)
Group	N	Mean	Mean ± SD	Mean ± SD	Mean ± SD
< 3 mg/m^3^·year	23	0.76	2.31 ± 0.97	9.24 ± 13.67	503.31 ± 1350.89
3–5 mg/m^3^·year	24	1.67	4.28 ± 2.05	42.43 ± 60.93	538.24 ± 735.64
5–12 mg/m^3^·year	26	2.41	5.31 ± 5.84	35.54 ± 59.26	542.01 ± 588.39
≥ 12 mg/m^3^·year	25	2.70	34.92 ± 60.37	462.75 ± 984.29	2037.62 ± 2493.50
Total	98	1.78	11.91 ± 33.11	140.04 ± 526.99	913.54 ± 1614.68

**Table 3:** Leukemia risk in benzene-exposed workers based on linearized multistage (LMS) modeling.

		Average CE	Leukemia risk (95% CI) × 10^−4^		
Group	N	mg/m^3^-year	by air benzene^#^	by S-PMA^‡^	by *t, t*-MA^§^
< 3 mg/m^3^·year	23	1.62	4.34 (4.34–4.35)	4.42 (4.38–4.45)	4.47 (4.35–4.58)
3–5 mg/m^3^·year	24	3.94	4.37 (4.37–4.38)	5.23 (4.89–5.56)	4.49 (4.41–4.56)
5–12 mg/m^3^·year	26	8.46	4.44 (4.43–4.44)	5.35 (5.05–5.65)	4.54 (4.51–4.58)
≥ 12 mg/m^3^·year	25	86.79	5.52 (4.98–6.05)	5.61 (2.91–8.31)	6.24 (5.83–6.64)
*p* value for trend*	-	-	<0.0001	0.36	0.0006
Total	98	25.73	4.67 (4.38–4.97)	18.05 (3.52–32.58)	4.94 (4.68–5.21)

*Test for trend was based on average leukemia risks for each group. CE: cumulative exposure, calculated by multiplying C_TWA_ and working duration; 95% CI: 95% confidence interval. #estimated from Equation (6); ‡estimated from Equation (9); §estimated from Equation (10).

Besides, it is noteworthy that among 5 individuals exhibited air benzene values were below the LOD, the median concentrations of S-PMA and *t, t*-MA were 1.79 μg/g Cr and 5.18 μg/g Cr, respectively. All of these 5 workers were non-smokers, and none of them had recently eaten foods containing preservatives (sorbic acid). It suggests that these 5 workers may have been exposed to low levels of benzene. The risk estimated from *t, t*-MA was found to be more similar to the leukemia risk derived from air benzene concentrations. Therefore, we used the *t, t*-MA to evaluate the leukemia risk among the 5 workers (Equation 10). The findings indicated that these 5 workers still face a potential risk of developing leukemia (leukemia risk value: 4.32 × 10^−4^).

### 3.4 Comparison of the results of the EPA model, the Singapore semi-quantitative risk assessment model and the LMS model

As shown in Table 4, the results of the three risk assessment models exhibited partial inconsistency. The Singapore semi-quantitative risk assessment model and the LMS model showed consistent results, indicating a high health risk. However, within the low concentration groups (< 3, 3–5 mg/m^3^·year), the EPA model estimated a lower leukemia risk compared to the LMS model. In contrast, in the high concentration groups (5–12, ≥12 mg/m^3^·year), both EPA and LMS models produced similar findings. It is noteworthy that all four groups exceeded the EPA’s lowest acceptable limit for carcinogenic risk, set at 1 × 10^−6^.

**Table 4:** Results of the three risk assessment models.

		C_TWA_	Average CE	Leukemia risk			
Group	N	mg/m^3^	mg/m^3^·year	LMS model	EPA model	Singapore semi-quantitative model	
						Risk value	Risk rating
< 3 mg/m^3^·year	23	2.31	1.62	4.34 × 10^−4^	(0.1 ~ 0.4) × 10^−4^	3.87	High risk
3–5 mg/m^3^·year	24	4.28	3.94	4.37 × 10^−4^	(0.3 ~ 1.2) × 10^−4^	3.87	High risk
5–12 mg/m^3^·year	26	5.31	8.46	4.44 × 10^−4^	(0.8 ~ 3.0) × 10^−4^	4.47	High risk
≥ 12 mg/m^3^·year	25	34.92	86.79	5.52 × 10^−4^	(6.1 ~ 21.7) × 10^−4^	5	Very high risk

## 4 Discussion

Several studies have been conducted to evaluate the leukemia risk associated with benzene using the LMS model, however, the findings have shown inconsistencies. Cox et al. (21) reported that there was no excess cancer risk at benzene concentrations below 3.25 mg/m^3^ by using the LMS model with estimated total benzene metabolites. Wang et al. (22) found a risk of 2.93 × 10^−5^ for workers exposed to benzene at 2.92 mg/m^3^ by using the LMS model with estimated blood benzene. Both total metabolites doses and blood benzene were used as internal dose of the LMS model. These internal doses were predicted using physiologically based pharmacokinetic models derived from animal experiments. To the best of our knowledge, this study is the first to use the LMS model for fitting epidemiological data on benzene and simultaneously employ passively monitored benzene concentrations as the external dose, and urinary benzene metabolites (*t, t*-MA and S-PMA) as the internal dose for leukemia risk assessment. Our results suggest that the excess risk of leukemia was 4.34 × 10^−4^ at low levels of benzene exposure (< 3 mg/m^3^·year; Table 3), and the risk increased with the cumulative concentration of benzene exposure. The excess risk of leukemia estimated in this study from low-level benzene exposure was similar to the leukemia risk estimated by multiple benzene cohorts (23, 24). Consistent with our findings, a case-cohort study also revealed a dose-dependent association between cumulative exposures (ranging from 0.13 to 3.08 mg/m^3^·years) and acute myeloid leukemia (6).

In the present study, the parameters (*r* and *a_i_*) of the LMS model were obtained from leukemia incidence data in a Chinese benzene cohort study. This large cohort study found that the leukemia risk increased with increasing exposure levels at cumulative exposure concentrations below 321 mg/m^3^·year. The benzene concentration was determined based on work histories and available historic benzene measurements, which were estimated by local industrial hygienists and occupational health personnel using detailed production and related process information, as well as airborne benzene measurements when available. Therefore, the estimated model parameters derived from this data can be considered relatively accurate.

In this study, the results of the LMS model were validated using both a quantitative risk assessment model (EPA model) and a semi-quantitative risk assessment model (Singapore semi-quantitative risk assessment model), respectively. The findings indicated that the risk assessment results of the LMS model were generally consistent with those of the Singapore semi-quantitative risk assessment model and similar to the EPA model at high concentrations (5–12, ≥12 mg/m^3^·year). At low concentrations of benzene exposure, the carcinogenic risk estimated by the EPA model was found to be comparatively lower than that by the LMS model [(0.1–0.4) × 10^−4^ and 4.34 × 10^−4^ for <3 mg/m^3^·year, respectively; and (0.3–1.2) × 10^−4^ and 4.37 × 10^−4^ for 3–5 mg/m^3^·year, respectively]. However, it still exceeded the lowest bound of the acceptable carcinogenic risk set by the EPA (1 × 10^−6^). The risk values assessed by the LMS model exhibited more accurate than those of the Singapore semi-quantitative risk assessment model, while for low concentrations of benzene, the risk values estimated by the LMS model exceeded the range of risks estimated by the EPA model. Our findings suggest that the LMS model is more sensitive in assessing the risk of leukemia among workers exposed to benzene, particularly at low levels of exposure.

Brett et al. (25) reported that the excess risk of leukemia was 5 × 10^−4^ after 45 years of exposure to benzene at 3.25 mg/m^3^ using data from the Pliofilm cohort study and employing the logistic regression method developed by Rinsky et al. (26). Using a proportional risk model, Paxton et al. (27) reanalyzed the cumulative benzene exposure in the Pliofilm cohort study, and estimated that the incidence of leukemia deaths among workers after 45 years of benzene exposure ranged from 0.3 to 0.5 per 1,000. The results of our study showed that workers in the <3 mg/m^3^·year group were exposed to an average concentration of 2.31 mg/m^3^ for 0.72 years (Table 2). Based on our assessment method, if they were able to work at this level of exposure for 45 years, their excess risk of leukemia would increase to 5.72 × 10^−4^, consistent with the aforementioned findings (5 × 10^−4^). Our findings suggest that the LMS model is suitable for evaluating the leukemia risk among workers exposed to low-levels of benzene.

There are also some limitations. First, the sample size of workers exposed to benzene was relatively limited. Further comprehensive investigations, utilizing a large number of samples, are needed to validate our findings. Second, extrapolating the absolute risk of leukemia at low-level benzene exposure based on the Chinese study conducted at high levels may underestimate the risk due to the observed non-linear relationship between leukemia risk and exposure levels exceeding 10 ppm in the cohort study (28). Third, the lack of using urinary cotinine is a limitation of our study. To account for the impact of smoking on benzene exposure, we only used smoking and non-smoking as binary categories. Since urinary cotinine serves as an equally effective biomarker as serum cotinine in indicating exposure to cigarette smoking (29), using urinary cotinine could accurately adjust for the confounding effect of smoking, particularly as it concerns the time since smoking the last cigarette before urine sampling.

Previous studies have reported that both urinary S-PMA and *t, t*-MA are reliable and specific biomarkers for assessing low-level benzene exposure (30). The estimated excess risk of leukemia, based on both air benzene concentration and urinary *t, t*-MA levels, exhibited a significant dose–response relationship (*P*_trend_ = 0.0006), with similar results. Notably, the LMS model estimation based on *t, t*-MA concentrations revealed that workers with air benzene concentrations below the limit of detection may still be at risk of developing leukemia. These findings suggest that urine *t, t*-MA might be more appropriate than air benzene in assessing the low-level risk of excessive leukemia. Further investigations involving larger cohorts of workers exposed to low levels of benzene are necessary to validate the findings presented in this study.

## 5 Conclusion

The findings of this study demonstrate the utility of the LMS model derived from the Chinese benzene cohort in assessing the leukemia risk associated with low-levels of benzene exposure. It should be noted that leukemia risk may occur at cumulative concentrations below 3 mg/m^3^·year.

## Data availability statement

The original contributions presented in the study are included in the article/supplementary material, further inquiries can be directed to the corresponding author.

## Ethics statement

The studies involving humans were approved by Committee for the Protection of Human Subjects at the National Institute for Occupational Health and Poison Control, Chinese Center for Disease Control and Prevention. The studies were conducted in accordance with the local legislation and institutional requirements. The participants provided their written informed consent to participate in this study.

## Author contributions

KJ: Writing – original draft. FZ: Writing – original draft. BW: Writing – review & editing. MinyL: Writing – review & editing. XW: Writing – review & editing. XC: Writing – review & editing. MingL: Writing – review & editing. DH: Writing – review & editing. CX: Writing – original draft.
